# Global assays and the management of oral anticoagulation

**DOI:** 10.1186/s12959-015-0037-1

**Published:** 2015-02-10

**Authors:** Herm Jan M Brinkman

**Affiliations:** Department of Plasma Proteins, Sanquin Research, Plesmanlaan 125, 1066 CX Amsterdam, The Netherlands

**Keywords:** PT, APTT, Thrombin generation, Thromboelastography, Apixaban, Rivaroxaban, Dabigatran, Vitamin K antagonists, Prothrombin complex concentrate, Anticoagulation reversal

## Abstract

Coagulation tests range from global or overall tests to assays specific to individual clotting factors and their inhibitors. Whether a particular test is influenced by an oral anticoagulant depends on the principle of the test and the type of oral anticoagulant. Knowledge on coagulation tests applicable in monitoring status and reversal of oral anticoagulation is a prerequisite when studying potential reversal agents or when managing anticoagulation in a clinical setting. Specialty tests based on the measurement of residual activated factor X (Xa) or thrombin activity, e.g., are highly effective for determining the concentration of the new generation direct factor Xa- and thrombin inhibitors, but these tests are unsuitable for the assessment of anticoagulation reversal by non-specific prohemostatic agents like prothrombin complex concentrate (PCC) and recombinant factor VIIa (FVIIa). Global coagulation assays, in this respect, seem more appropriate. This review evaluates the current status on the applicability of the global coagulation assays PT, APTT, thrombin generation and thromboelastography in the management of oral anticoagulation by vitamin K antagonists and the direct factor Xa and thrombin inhibitors. Although all global tests are influenced by both types of anticoagulants, not all tests are useful for monitoring anticoagulation and reversal thereof. Many (pre)analytical conditions are of influence on the assay readout, including the oral anticoagulant itself, the concentration of assay reagents and the presence of other elements like platelets and blood cells. Assay standardization, therefore, remains an issue of importance.

## Introduction

With the introduction in the 1940’s of vitamin K antagonists (VKAs) as an oral anticoagulant drug for the treatment of patients at risk for a thromboembolic event, the need for proper coagulation testing emerged [1,2]. In the early days of anticoagulant drug development, coagulation was a simple 4-factor mechanism consisting of thromboplastin, calcium, fibrinogen and prothrombin [3]. The prothrombin time assay introduced by Quick was performed in plasma taken from blood collected into sodium oxalate and clotting was initiated by adding calcium and thromboplastin reagent (crude tissue factor extract) from rabbit brain [4]. Owren, with the discovery of the clotting factors V, VII, VIII, IX, X, XI and XII, introduced a mixture of thromboplastin, cephalin (unrefined lipid extract containing phosphatidylethanolamine and phosphatidylserine) and aluminum hydroxide-absorbed plasma in order to make the assay more sensitive to anticoagulant treatment with VKAs [5]. Both methods, albeit with better defined reagents, are still widely recommended in guidelines on the management of oral anticoagulation by VKAs [6-8].

The development of coagulation tests goes hand in hand with increasing knowledge on the coagulation system. Evolving clinical experience has made practitioners doubting the value of the PT test in the management of VKA anticoagulation [9-11]. Also, the introduction of a new class of oral anticoagulants that target a specific activated clotting factor requires re-evaluation of the usefulness of the PT in the management of oral anticoagulation. Recent guidelines already suggest the use of thromboelastography in the management of VKA anticoagulation [12]. Thrombography, for which point of care tests are currently being developed, will soon follow [13]. However, these assays are complex and therefore should be introduced in general practice with caution. Knowledge on the assay principles as well as on the mechanism of action of the anticoagulant and its reversal agent is inevitable related to an adequate use of global assays in anticoagulation management. The wide variety of global assay and reagents available underscores the need for standardization and assay validation. In this review, a comparison is made between VKAs and direct thrombin and factor Xa inhibitors with respect to assay sensitivity and laboratory monitoring options for the control of anticoagulation reversal by non-specific hemostatic agents.

### Oral anticoagulants and reversal agents

#### Vitamin K antagonists

**Figure 1:**
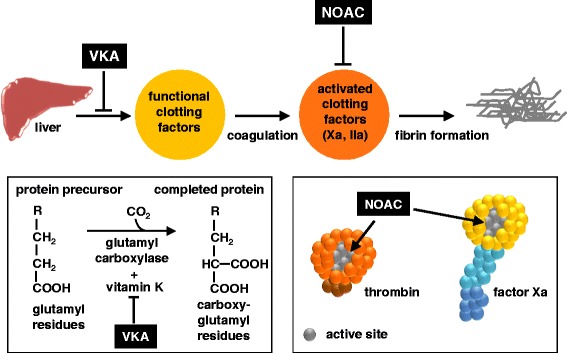
**Oral anticoagulation; mechanism and site of action.**

The history of oral anticoagulation starts in 1939 with the isolation of Dicoumarol. This drug became the prototype of a variety of orally administered coumarin derivatives with anticoagulant properties such as warfarin and phenprocoumon [14-16]. Coumarins, also known as vitamin K antagonists (VKAs), act by inhibiting the enzyme vitamin K reductase. During the post-translational carboxylation of vitamin K-dependent procoagulant factors II, VII, IX, X, as well as the natural vitamin K-dependent anticoagulant protein C and protein S by gamma-glutamyl-carboxylase, vitamin K removes hydrogen atoms from glutamic acid residues. Vitamin K than collapses into vitamin K epoxide and is recycled back to active vitamin K following the action of vitamin K reductase [17]. By inhibiting vitamin K reductase, the carboxylation process is downscaled. This results in the synthesis of vitamin K-dependent clotting factors with fewer or no gamma-carboxy-glutamic-acid (Gla) residues and hence with severely hampered binding properties to negatively charged surfaces (Figure 1). Binding of vitamin K-dependent clotting factors to negatively charged phospholipids is a necessity to facilitate hemostasis [18]. VKAs are anticoagulant because they suppress the synthesis of functional membrane-binding clotting factors.

#### Reversal of vitamin K antagonist-induced anticoagulation

A major complication with the use of VKAs is bleeding. The widespread use of VKAs in clinical practice, therefore, is just a matter of statistics: the number of people that is protected from major thrombotic complications is greater than the number of people showing VKA-associated bleeds [19]. Furthermore, clinicians have VKA-reversal agents at their disposal. First in line is vitamin K, suppressing the action of coumarins. De-novo synthesis of vitamin K-dependent clotting factors, however, may take too long. For immediate emergency reversal, replenishment of functional vitamin K-dependent clotting factors seem more appropriate [6,7]. This can be achieved by intravenous administration of 4-factor prothrombin complex concentrate (PCC), consisting of plasma derived human prothrombin, factor VII, factor IX and factor X. It should be noted that most PCCs also contain the vitamin K-dependent coagulation inhibitors protein C and protein S and in addition are supplemented with antithrombin and/or heparin [20]. The use of fresh frozen plasma, three-factor PCC (lacking factor VII) and recombinant factor VIIa as reversal agents for VKA may also be considered but their use is not encouraged [6,7].

#### Non-vitamin K antagonist oral anticoagulants

When using VKAs and apart from the increased bleeding risk, the following drawbacks need to be considered: slow onset and slow offset, more than 120 known food and drug interactions, requirement for regular monitoring [21]. These disadvantages of VKAs has led to the development of oral anticoagulant drugs that directly target activated factor X and thrombin (Figure 1). These novel oral anticoagulants (NOACs), also addressed as direct oral anticoagulants (DOACs), target specific oral anticoagulants (TSOAs) or non-vitamin K antagonist oral anticoagulants (NOACs), are small synthetic compounds that reversibly bind to the active site of factor Xa or thrombin [22-27]. To date, three NOACs have been approved for use in specific patients groups: the factor Xa inhibitors apixaban and rivaroxaban and the thrombin inhibitor dabigatran [28].

#### Reversal of non-vitamin K antagonist oral anticoagulation

Reversal agents that specifically target NOACs are under development and presently unavailable for general clinical use [29,30]. Current guidelines unanimously suggest the use of PCC as first in line drug in emergency situations with direct factor Xa inhibitor-associated bleeds but these guidelines are contradictory with regard to reversal of anticoagulation by dabigatran [8,12,28,31]. Recombinant activated factor VII (rFVII) and activated factor VII-containing PCC (activated PCC), agents that are effective in the therapy of bleeding episodes in hemophilic patients with inhibitors, may also be of potential use [8,12,28,31]. Mechanism of action of PCC in the reversal of NOAC anticoagulation differs from that in VKA reversal. With respect to VKA reversal, PCC replenishes the level of functional vitamin K dependent clotting factors. With regard to NOAC reversal, functional clotting factors already are present and reversal of NOAC anticoagulation by PCC most likely is due to an increased number of factor Xa or thrombin molecules escaping from inhibition [32]. A similar model may also be applicable for rFVIIa and activated PCC, clotting factor concentrates just as PCC able to increase thrombin generation when added to normal plasma [33,34]. In addition, rFVII may also improve platelet deposition at sites of vessel trauma [35].

### Global coagulation assays

#### Clotting time

**Figure 2:**
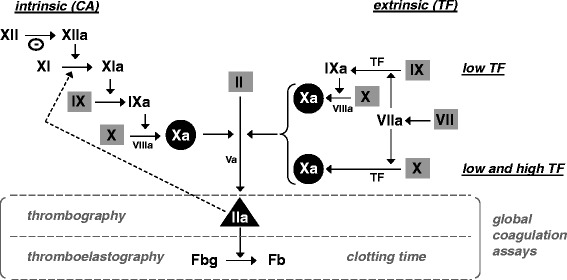
**Coagulation pathways implicated in intrinsic (contact activation, CA) - and extrinsic (tissue factor, TF) based global assays with highlighted targets for oral anticoagulation.** Cofactors V and VIII are indicated in their activated forms. Grey squares indicate vitamin K-dependent procoagulant factors, targets for VKA anticoagulation. Black circles indicate the target for direct factor Xa inhibitors, i.e. rivaroxaban and apixaban. Black triangle shows the target for the direct thrombin inhibitor dabigatran. Specificity and sensitivity of extrinsic pathway based assays is determined by the tissue factor (TF) concentration. At high TF concentrations, enough factor X is activated directly by TF/VIIa and hence the contribution of factor IX and factor VIII is negligible. At low TF concentrations (<5 pM), extrinsic based assays are also sensitive to factor VIII and IX. Dotted arrowed line indicates feedback activation of factor XI by thrombin (IIa), allowing factor XI to be involved in the extrinsic pathway but this is noticed only at very low tissue factor concentration (≤1 pM). Θ, negatively charged surface, e.g. celite or kaolin, facilitating contact activation. Fbg, fibrinogen. Fb, fibrin.

Global tests, in contrast to assays specific to individual clotting factors, provide an overall assessment of the functioning of the coagulation system. Coagulation in global tests is triggered either by agents that contain negatively charged particulate (kaolin, silica, ellagic acid, celite) to initiate contact activation (intrinsic pathway), or by reagents that contain tissue factor (TF) to initiate the extrinsic pathway (Figure 2). The most widely used global coagulation test is the clotting time. Triggered with TF, this assay is known as the prothrombin time (PT), while the variant with contact activation triggered coagulation is known as the activated partial thromboplastin time (APTT). This nomenclature is from historical origin. The PT test was developed in the beginning of the last century when all clotting factors except prothrombin still had to be discovered. Triggering reagent is thromboplastin, a term originally used to describe a substance in plasma that converts prothrombin to thrombin [4]. Historically, thromboplastins were extracted from brain and other organs and these extracts contained significant amounts of TF and phospholipid. The term “partial” in APTT refers to reagents without TF, while “activated” refers to the use of negatively charged particulate contact activators to improve responsiveness and reproducibility [36].

**Figure 3:**
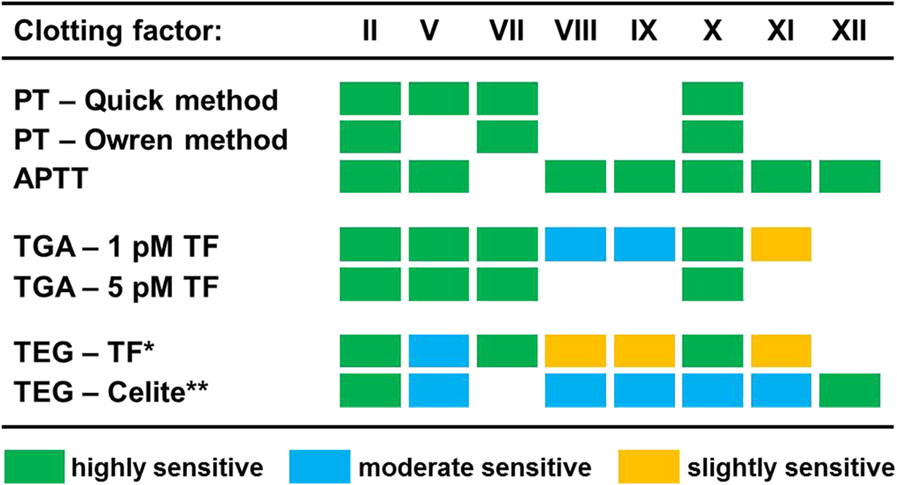
**Clotting factor sensitivity of different global coagulation assays.** This figure shows the sensitivity of global tests for clotting factor deficiencies. A mark indicates that an assay is highly sensitive (green), moderate sensitive (blue), or slightly sensitive (orange) to the absence (<1%) of a certain clotting factor. No mark indicates no sensitivity, i.e. a normal assay readout. Sensitivity of different global coagulation tests for deficiencies in procoagulant factors are based on data provided in the following publications: clotting time based PT and APTT [38,39], thrombin generation assay (TGA) [40], thromboelastography (TEG) [41]. The presence of factor V in the assay reagents makes the Owren PT insensitive to this coagulation factor. *0.1% rabbit brain thromboplastin. **Negatively charged particulate to initiate contact activation.

The PT and APTT are performed with citrated platelet poor plasma to which calcium and trigger reagent is added. With these tests, the time is recorded until a visible clot is formed. In the PT according to the Quick method [4] as well in the APTT, undiluted test plasma is used. In the PT following the Owren method [5], test plasma is diluted with absorbed bovine plasma as source of factor V and fibrinogen. A wide variety of thromboplastins with inconsistent clotting activity has led to the introduction of the international normalized ratio (INR) that aims to harmonize PT results obtained with VKA-anticoagulated plasma regardless of the reagent and instrument used [37]. The APTT is sensitive to deficiencies in all clotting factors implicated in the intrinsic pathway (Figures 2 and 3). Clotting factor sensitivity of the PT by design is downscaled to fibrinogen and the factors II (prothrombin), V, VII and X. Due to high TF concentrations in the triggering reagent, the PT is insensitive to factor VIII and factor IX (Figures 2 and 3). The Owren method, in contrast to the Quick method, is also insensitive to fibrinogen and factor V, as these compounds are present in the supplied reagents.

Drawback of the PT and APTT is that it measures the clotting time only. Once a visible clot is formed, thrombin and fibrin formation proceeds until a clot with maximal firmness is produced and the coagulation process is inhibited [42,43]. PT and APTT thus do not record processes beyond initial clotting. Another drawback of the PT and APTT is the lack of cellular contributions to fibrin network formation [44]. These drawbacks are challenged by more advanced global assays including thromboelastography/thromboelastometry and the thrombin generation assay.

#### Thromboelastography/thromboelastometry

Thromboelastography (TEG) or thromboelastometry (TEM) measures the mechanical resistance of an indicator rod in clotting whole blood or plasma. Depending on the type of equipment, either the indicator rod (ROTEM) or the cup containing the whole blood or plasma (TEG) is continuously twisting left and right during analysis. As a consequence of fibrin formation, viscoelasticity of the whole blood or plasma will increase in time with concomitant increase in mechanical friction on the indicator rod. Rephrased, thromboelastography measures the formation (and degradation) of a fibrin clot in time in whole blood or plasma. Parameters derived from TEG/TEM tracings include reaction time (R) or clotting time (CT) defined as the period to 2 mm amplitude, kinetics (K) or clot formation time (CFT) being the period from 2–20 mm amplitude, angle (A) being the slope of the tracing, and maximum amplitude (MA) or maximum clot firmness (MCF) [45]. With this technique, both intrinsic and extrinsic coagulation triggers can be applied (Figures 2 and 3).

#### Thrombin generation assay

Of enormous edifying value is the measurement of active thrombin in clotting plasma over time. This technique is called thrombography and utilizes thrombin sensitive fluorogenic or chromogenic peptide substrates [46]. These synthetic substrates, however, are cleaved by both free thrombin and alpha-2-macroglobulin bound thrombin, as such overestimating the thrombin generating potential of the plasma sample [47]. The algorithm that is used in the calibrated automated thrombography (CAT) method, corrects for the activity of alpha-2-macroglobulin bound thrombin [48,49]. Advantage of fluorogenic substrates over chromogenic substrates is that inhibition of fibrin polymerization is not required. The thrombin generation assay (TGA) is flexible by design and allows modifications with respect to coagulation triggering reagents, buffers, additives and the presence of vascular cells and platelets. Of innovative importance is thrombin generation in whole blood [13]. Parameters derived from thrombography include lag time, peak height and area under the curve (AUC) or extrinsic thrombin potential (ETP).

### Effect of old and novel oral anticoagulants on global assays

#### Influence of VKAs on global assays

VKA anticoagulation will affect any global assay that is dependent on functional vitamin K-dependent coagulation factors (Figure 2). Influence of VKAs on global assays, therefore, is not restricted to the for VKA monitoring generally applied PT test. Outcome of APTT, TGA and TEG/TEM testings are affected by VKAs as well (Table 1). Sensitivity of the different global tests, however, is greatly dependent on the coagulation trigger of choice as well as on the trigger concentration. E.g., the contact activation-triggered APTT, in general, is less sensitive to VKA treatment than the TF-triggered PT test [50,51]. Whole blood point of care PT devices may generate a slightly increased outcome as compared to standard laboratory PT assays in plasma, a phenomenon that may relate to the chemistry used (Owren or Quick based) and the presence of blood cells and platelets [52-54].

**Table 1:** **At a glance: global assay response to anticoagulation treatment**

**Assay**	**Parameter**	**Oral anticoagulant**			
		**VKA**	**Apixaban**	**Rivaroxaban**	**Dabigatran**
PT-Quick	Clotting time	↑	−	↑	↑
PT-Owren	Clotting time	↑	−	↑	↑
APTT	Clotting time	↑	−	↑	↑
TGA-TF	Lag time	↑	↑	↑	↑
	Peak	↓	↓	↓	↓
	AUC	↓	↓	↓	↓
TEG/TEM-TF	R/CT	↑	↑/−	↑	↑
	K/CFT	↑	−	−	−
	MA/MCF	↓	−	−	−
TEG/TEM-CA*	R/CT	↑/−	↑/−	↑	↑
	K/CFT	−	−	−	−
	MA/MCF	−	−	−	↓/−

Qualitative comparison of the effect of NOACs on global testing parameters. PT, APTT and TGA assays were performed in citrated plasma. TEG/TEM assays were performed in citrated whole blood. Assay parameter is significantly increased (↑) or decreased (↓) by the oral anticoagulant, or effect is marginal to unnoticed (−). Classifications are based on the following publications: VKA [55-59], apixaban [32,35,60-63], rivaroxaban [62-69], dabigatran [32,56,63,69-74]. *Contact activator kaolin or celite. Global assays are sensitive to oral anticoagulation by VKAs, direct factor Xa inhibitors and direct thrombin inhibitors. Global coagulation assays, in general, do not show specificity to a particular drug. They are only different in drug sensitivity (see Table 2).

**Figure 4:**
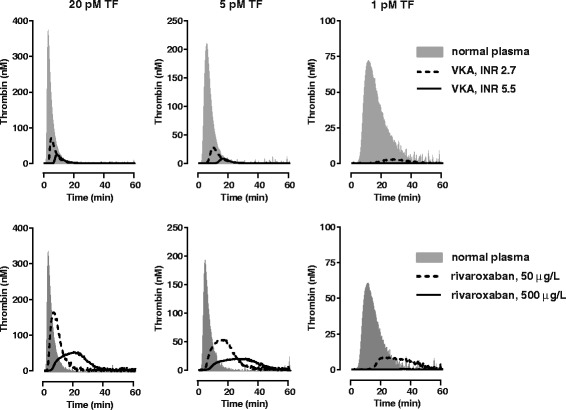
**Influence of TF concentration on the thrombographic assessment of oral anticagulation.** Thrombin generation with VKA anticoagulated plasma (George King Biomedical Inc, Overland Park, Kansas, USA ) and rivaroxaban-spiked normal plasma was performed with triggering reagent containing 4 μM phospholipids and 1, 5, and 20 pM TF as described [68]. At decreasing TF, thrombin generation decreases; i.e. longer lag time and decreased peak height and AUC. At a TF concentration of 1 pM, thrombin generation in VKA- and rivaroxaban anticoagulated plasma is unnoticed at high anticoagulant levels, while detectable at 5 and 20 pM. Reversal of oral anticoagulation can be achieved, e.g., by PCC (see Figure 6).

An example of the effect of different TF concentrations on the thrombographic analysis of VKA-anticoagulated plasma is shown in Figure 4. At increasing TF, thrombin generation increases; i.e. shorter lag time and increased peak height and AUC. At a TF concentration of 1 pM, thrombin generation in VKA-anticoagulated plasma is often unnoticed while detectable at 5 and 20 pM. In TGA, a TF concentration of 5 pM or higher is generally practiced when monitoring VKA anticoagulation [55,56,75]. The TF concentration in the for management of VKA-anticoagulation commonly used PT test is much higher (>1 nM). Major advantage of using a low 1 pM concentration of TF is the gained or increased sensitivity to factor IX, protein Z and tissue factor pathway inhibitor (TFPI), proteins to be held responsible for the failure of the INR to adequately reflect the anticoagulant state in some individuals on VKA [76-78]. Of importance is whether thrombomodulin (TM) is present during analysis. TM is a component of the vascular wall and essential for the generation of activated protein C and concomitant functioning of the protein C/S anticoagulant pathway. For TGA it has been concluded that in the absence of TM, thrombin generation in VKA anticoagulated plasma is overestimated [79].

Controversial data have been reported regarding the applicability of the TEG/TEM in monitoring VKA treatment. Reports have shown very poor sensitivity of the whole blood TEG towards VKA treatment and TEG outcome was normal in a considerable amount of VKA patients despite an increased PT (INR 1.5-2.8) [57,58]. In a study among healthy volunteers, however, both PT/INR and TEG readout was substantially altered upon VKA treatment [59].

In summary, the PT/INR remains the test of choice for monitoring VKA anticoagulation. APTT in general is less sensitive to VKA treatment than the commonly applied PT and is not recommended. Applicability of the TEG/TEM in the management of oral anticoagulation by VKAs is questionable and extensive validation is required before adding this technique to general guidelines. TGA is promising but requires validation and standardization.

#### Influence of NOACs on global assays

**Figure 5:**
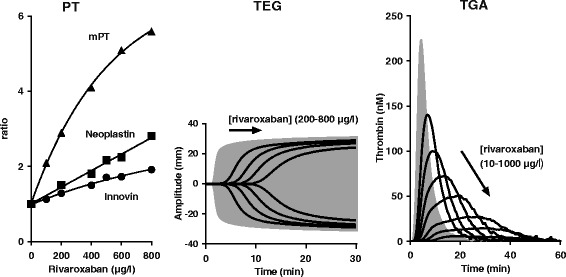
**Influence of rivaroxaban on PT, TEG and TGA.** Normal plasma spiked with increasing rivaroxaban concentrations was subjected to PT, TEG and TGA measurements as described [32,68]. PT reagents Neoplastin and Innovin are commercially available from Diagnostica Stago (Asnieres sur Seine, France) and Siemens Healtcare Diagnostics (Marburg, Germany) respectively. The modified PT (mPT) reagent was prepared by mixing 1 volume Thromborel S (Siemens Healthcare Diagnostics) with 1.25 volumes 80 mM CaCl_2_. TEG was with 4 μM phospholipids (Rossix AB, Mölndal, Sweden) and 10 pM TF (Innovin, Diagnostica Stago). TGA was with the CAT reagents from Thrombinoscope (Maastricht, The Netherlands) and includes the PPP reagent (4 μM phospholipids/5 pM TF). TEG and TGA filled grey curves: normal plasma, solid black lines: increasing dabigatran concentration. Correlations between rivaroxaban dose and assay outcome were used to calculate the effective rivaroxaban concentration in a particular test. The mPT, TEG-R and TGA-lag time appeared most sensitive to rivaroxaban (see Table 2).

NOACs may affect any assay that depends on factor Xa or IIa (thrombin) activity, including the PT, APTT, TGA and TEG/TEM (Figure 2). Effect of NOACs on global tests have been shown in several publications [32,60-68,70-72,80]. However, experimental conditions, used tests and reported output parameters vary between the different studies, allowing only a qualitative comparison of published data between different NOACs and the different global tests (Table 1). A detailed, quantitative in vitro comparison between the effect of apixaban, rivaroxaban and dabigatran on different global tests performed with platelet poor plasma under identical experimental conditions is shown in Table 2. For rivaroxaban, examples showing dose–response relationships in PT, TGA and TEG are shown in Figure 5.

**Table 2:** **In detail: NOAC sensitivity of different global coagulation tests in plasma**

		**Rivaroxaban**	**Apixaban**	**Dabigatran**
		*In vivo* therapeutic dose^1^		
	Acute VTE:	15 mg bid	10 mg bid	150 mg bid
	Prophylaxis:	20 mg od	5 mg bid	150 mg bid
		*In vivo* mean plasma concentration (C_min_-C_max_, μg/L)^1^		
	Acute VTE:	100 - 270	104 - 330	93 - 184
	Prophylaxis:	45 - 250	50 - 128	93 - 184
		*In vitro* effective concentration (μg/L)^2^		
PT	– Innovin	399 ± 49	>800	596 ± 73
	– Thromborel	392 ± 36	>800	554 ± 41
	– Neoplastin	214 ± 36	>800	538 ± 47
modified PT	– mPT-Innovin	43 ± 3	190 ± 13	64 ± 6
	– mPT-Thromborel	47 ± 3	80 ± 6	88 ± 10
APTT	– Actin FSL	254 ± 28	>800	190 ± 15
TGA	– Lag time	41 ± 5	93 ± 28	27 ± 7
	– Peak thrombin	109 ± 5	121 ± 4	380 ± 71
	– AUC	151 ± 36	327 ± 99	433 ± 71
TEG-TF	– R	28 ± 3	80 ± 17	16 ± 8
	– Angle	263 ± 66	721 ± 73	484 ± 3
	– MA	>800	>800	>800

^**1**^NOAC dose (od, once daily; bid, twice daily) currently advised for the treatment of acute venous thromboembolism (VTE) and the prophylactic treatment of VTE and atrial fibrillation [81,82] with mean NOAC concentration in plasma at steady state during treatment pre dose (C_min_) and 2 h post dose (C_max_) [83-85].

^**2**^Pooled normal citrated plasma was spiked with NOACs ranging from 0–800 μg/L plasma and subjected to PT APTT, TGA and TEG analysis. The modified PT (mPT) reagent consisted of a mixture of 1 volume thromboplastin reagent and 1.25 volumes 80 mM CaCl_2_ [85]. The TGA assay was with 5 pM TF and 4 μM phospholipids. The TEG-TF in plasma was with 10 pM TF and 4 μM phospholipids. Effective concentration (EC_±50%_) was defined as a 50% increase or decrease in assay parameter by the NOAC of interest as compared to incubations without NOAC. EC_±50%_ values were obtained by interpolation and are given as mean ± SD of at least 3 determinations with the same plasma pool. Part of the data were taken from Dinkelaar et al. and detailed methods can be found in that study [32].

PT and APTT, as well as other global tests, do not show specificity to a particular drug. They are only different in drug sensitivity. PT and APTT show poor sensitivity to apixaban, while significantly affected by rivaroxaban and dabigatran (Table 2). For these two drugs, concentrations >200 μg/L were required to increase the clotting time by 50%. This suggests that a significant change in PT and APTT is only achieved at relatively high drug levels. Indeed, PT and APTT are often normal in patients on therapeutic doses of rivaroxaban and dabigatran [86,87]. Of importance is the extent by which the plasma sample is diluted when performing a PT; typically 3 fold with the Quick method and 20 fold when performing a PT according to the method of Owren. A more diluted sample with the Owren method will result in lower NOAC levels during PT measurements. The PT test according to Owren, therefore, is often less sensitive to NOACs as compared to the Quick method [61,65,88-90]. Strongly approved sensitivity with an effective concentration within the clinical therapeutic dose range for all NOACs, including apixaban, was observed with a modified PT (mPT) reagent consisting of thromboplastin diluted with CaCl_2_ (Table 2, Figure 5). The non-linear dose–response relationship as observed for rivaroxaban with mPT but also with standard PT reagents, is usually much less prominent for apixaban and dabigatran. Another issue of importance is standardization, given the high variability in NOAC response between different thromboplastin reagents (Table 2 [61,64-66,70,88,90,91]), an essential aspect when applying the PT test to NOAC monitoring. For VKA anticoagulation, it is general practice to normalize PT outcome to INR using an international sensitivity index (ISI) supplied by the manufacturer of the used thromboplastin reagent. ISI values for VKA-anticoagulated plasma, however, dramatically magnifies the between-thromboplastin variability in response to NOACs and thus do not apply to NOAC-anticoagulated plasma [90-92]. For each NOAC, separate ISI values need to be established [93,94].

Explaining the effect of NOACs on TGA requires some background information regarding coagulation pathways. During the initiation phase of coagulation, thrombin generation is primarily dependent on the concentration of the TF/FVIIa complex [95] and thus on feedback activation of factor VII by coagulation proteases including factor Xa and thrombin [96]. During the propagation phase, in which the bulk of thrombin is generated, thrombin generation is predominantly dependent on the concentration of factor Xa [95]. This might suggest that direct Xa inhibitors affect both initiation phase (TGA lag time) and propagation phase (TGA peak and AUC) while direct thrombin inhibitors only affect the initiation phase. On the other hand, direct thrombin inhibitors will inhibit feedback activation of factors V and VII in the initiation phase, thereby determining the amount of factor Va/Xa complexes available for thrombin generation in the propagation phase. In TGA, therefore, all parameters are affected by direct factor Xa inhibitors as well as by direct thrombin inhibitors (Tables 1 and 2).

Complicating factor in TGA is that direct thrombin inhibitors not only interact with free thrombin, but also with thrombin in complex with alpha-2-macroglobulin. The CAT method corrects for the activity of alpha-2-macroglobulin-bound thrombin, but the used algorithm does not take into account that thrombin bound to alpha-2 macroglobulin also is inhibited. This results in a small (±10%) but significant, albeit artificial, increase in AUC and thrombin peak at low (<100 nM) plasma concentrations of a direct thrombin inhibitor when applying this method [97]. Lag time does not show this artifact. Direct thrombin inhibitor-induced hypercoagulability has also been noticed as the consequence of reduced protein C anticoagulation, a feature predominantly observed in the presence of thrombomodulin [69,98]. One should also be aware of the fact that with CAT, the calibrator (alpha-2-macroglobulin-thrombin complex) also is inhibited by direct thrombin inhibitors. For plasma samples that contain a direct thrombin inhibitor, it is advisable, therefore, to use normal plasma for calibration.

A major determinant of the NOAC effect in TF-triggered assays such as the TGA is the tissue factor concentration (Figure 4) [32,56,68]. At high TF (>5 pM), maximal levels of factor Xa and thrombin are generated with significant number of factor Xa or thrombin molecules escaping from inhibition by NOACs. At low TF (<5 pM), thrombin generation is tempered with probably less factor Xa or thrombin molecules escaping from NOAC inhibition.

TEG in platelet poor plasma and triggered with 10 pM TF showed responsiveness of the output parameters R-time and angle to rivaroxaban, apixaban, as well as dabigatran. Maximal amplitude was not affected by the NOACs. R-time was the most sensitive parameter, revealing effectiveness in the therapeutic dose range for all three NOACs (Table 2). In the whole blood TEM with standard reagents (EXTEM, INTEM), only the clotting time is affected to some extent [32]. When applying in a clinical setting, a modified whole blood TEG/TEM with very low TF or a TEG/TEM without TF or kaolin/celite seems more appropriate but this requires further validation [64,72,99]. Of importance is the notion that whole blood assays are affected by NOACs to a lesser extent than assays in platelet poor plasma. E.g., the dabigatran dose needed to double R-time in TEG triggered with 10 pM TF was 43 μg/l in platelet poor plasma as compared to 187 μg/l in whole blood [32]. A similar observation was made with TEG for apixaban [32] and for rivaroxaban in TGA [68].

Thus, although all global assays are affected by all NOACs (Table 2), applicability of these tests in monitoring NOAC treatment is limited. Due to low assay sensitivity, the PT, APTT, TGA-peak, TGA-AUC and TEG/TEM-angle may only be suitable for detecting anticoagulation at supratherapeutic NOAC plasma levels. The mPT, TGA-lag time and TEG-R, assayed in platelet poor plasma, may be the only generally applicable parameters in clinical practice when anticoagulation monitoring is required, but this needs further exploration.

### Global assays and procoagulant treatment

#### Assessment of anticoagulation reversal

**Figure 6:**
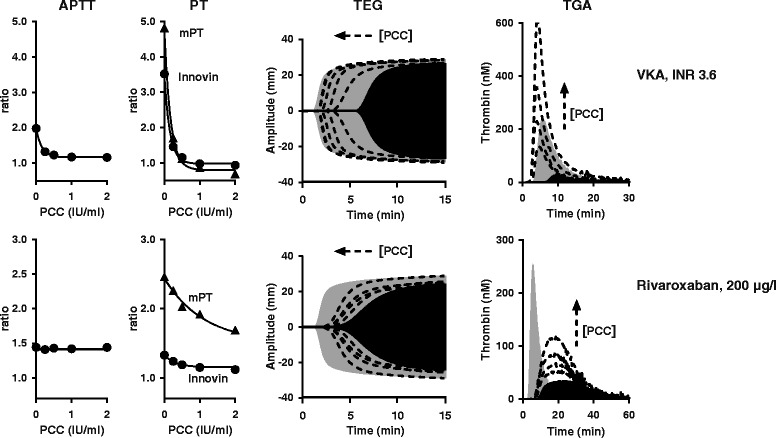
**In vitro reversal of VKA- and rivaroxaban anticoagulation by PCC.** VKA anticoagulated plasma (George King Biomedical Inc) and normal plasma anticoagulated with 200 μg/L rivaroxaban was spiked with increasing PCC dose (4-factor PCC, Cofact, Sanquin, Amsterdam, The Netherlands). APTT, PT, TEG and TGA was performed as described [32,68]. APTT was with reagent Actin FSL from Siemens Healthcare Diagnostics. PT was with Innovin (Siemens). The modified PT (mPT) reagent was prepared by mixing 1 volume Thromborel S (Siemens Healthcare Diagnostics) with 1.25 volumes 80 mM CaCl_2_. TEG was with 4 μM phospholipids (Rossix AB) and 10 pM TF (Innovin, Diagnostica Stago). TGA was with 4 μM phospholipids and 5 pM TF (PPP reagent, Thrombinoscope). Filled grey TGA and TEG curves: normal plasma, filled black curves: anticoagulated plasma without spiked PCC, dotted lines: anticoagulated plasma with increasing PCC dose (0, 0.25, 0.5, 1, 2 IU/ml). Remarkable feature for NOAC reversal is that the response to PCC is strongly TF concentration dependent; at a high TF concentration, less PCC is needed to restore TGA-peak and TGA-AUC [32,68]. 1 IU/ml PCC ≈ 40 IU per kg body weight.

**Figure 7:**
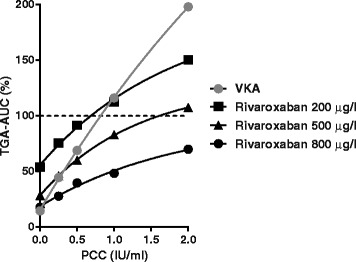
**Monitoring in vitro reversal of VKA- and rivaroxaban anticoagulation with TGA-AUC.** VKA plasma (INR 3.6, George King Biomedical Inc) and rivaroxaban-anticoagulated plasma spiked with increasing PCC dose (4-factor PCC, Cofact, Sanquin) was subjected to TGA (4 μM phospholipids and 5 pM TF as described [32,68]. TGA-AUC is expressed as % of not anticoagulated normal plasma without PCC. Correlation between PCC and TGA-AUC is more or less linear for VKA while less steeper and decaying for rivaroxaban. For apixaban and dabigatran, similar decaying curves were observed [32]. A decaying curve results in PCC incapable in restoring TGA-AUC to normal at high to extreme NOAC levels. This figure also shows that the suggested PCC dose for treatment of rivaroxaban-associated bleeds of 50 IU per kg body weight (±1.25 IU/ml) [12] is able to fully normalize the TGA-AUC at 200 μg/L rivaroxaban and to achieve almost complete TGA-AUC normalization at 500 μg/L.

As some global assay parameters show good responsiveness to oral anticoagulants, these parameters may be useful in the assessment of anticoagulation reversal. From a historical perspective, oral anticoagulation as well as the effect of reversal agents is monitored by PT. For VKA anticoagulation this might be valid. However, the introduction of NOACs requires the re-evaluation of currently applicable global assays. In Figure 6, the reversal of VKA anticoagulation (INR 3.6) by PCC is compared with that for rivaroxaban (200 μg/l) in PT, APTT, TGA and TEG. At first glance it can be observed that in all assays VKA anticoagulation is completely reversed by PCC, while the reversal effect of PCC on rivaroxaban anticoagulation is less pronounced. The mPT, TGA-lag time and TEG-R, parameters showing sensitivity in the therapeutic dose range for all NAOCs (Table 2), do not show complete correction by PCC. The only assay parameter for which complete normalization of rivaroxaban (200 μg/l) anticoagulation by PCC was observed was the TGA-AUC (Figure 7).

**Figure 8:**
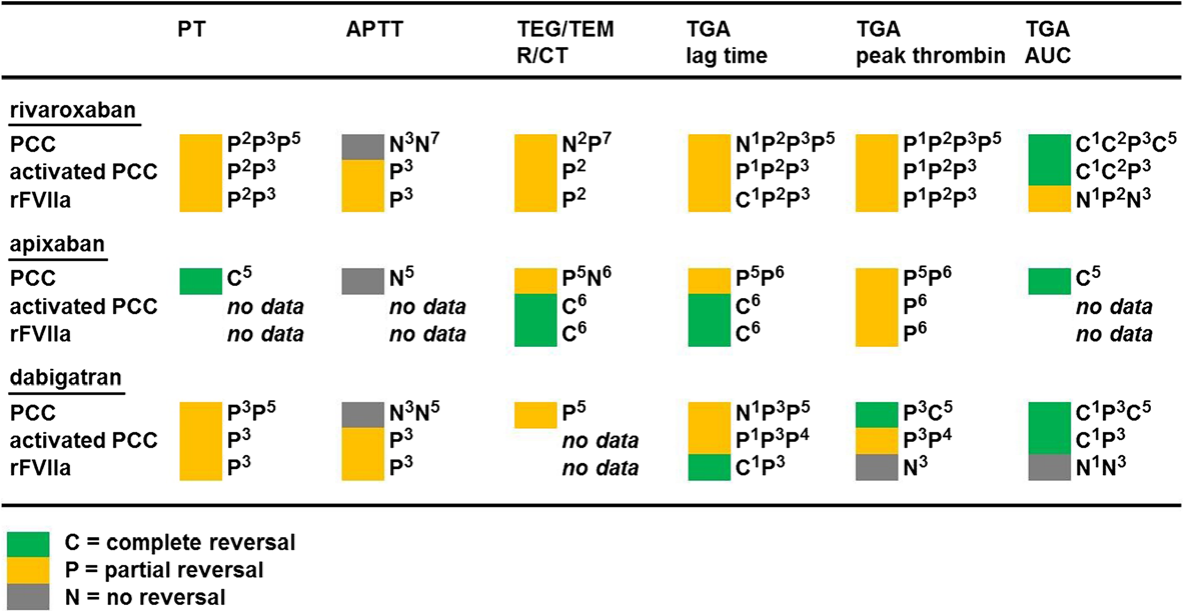
**Summary of in vitro NOAC reversal data with PCC, activated PCC and rFVIIa.** Data on in vitro NOAC reversal in human whole blood or plasma with APTT, PT, TEG/TEM (TF-triggered) and TGA (TF-triggered) were from the following studies: ^1^Marlu et al. [101], ex vivo reversal with plasma from NOAC treated healthy volunteers. ^2^Perzborn et al. [100], in vitro spiking experiments. ^3^Herrmann et al. [102], ex vivo reversal with plasma and whole blood from NOAC treated patients. ^4^Khoo et al. [103], ex vivo reversal with plasma from NOAC treated patients. ^5^Dinkelaar et al. [32,68], in vitro spiking experiments. ^6^Escolar et al. [35], in vitro spiking experiments. ^7^Additional in vitro data presented in this review. Ex vivo reversal studies with NOAC-treated patients are difficult to interpret due to lack of a reliable reference point, i.e. plasma from the same patient not treated with NOAC. Ex vivo reversal in patients, if present, is therefore classified as partial. In the overall classification (colored marks), in vitro spiking studies and ex vivo reversal studies with healthy volunteers are dominators. Most readout parameters only show partial reversal of anticoagulation.

In VKA-anticoagulated plasma, complete correction of all global assay readout parameters by PCC as observed in Figure 6 was expected to take place on the basis of Figure 2 due to replenishment of functional vitamin K-dependent coagulation factors. Mechanisms implicated in the influence of clotting factor concentrates on global assay readout parameters in NOAC anticoagulated plasma, however, are complex and difficult to predict (discussed in: [32]). Carefully performed feasibility studies, therefore, are essential before applying global assays in clinical practice. In vitro spiking experiments, in which both NOAC and reversal agent are added to normal whole blood or plasma in a controlled setting, as in Figure 6, are ideal for this purpose [32,35,68,100]. Ex vivo reversal studies, using whole blood or plasma from anticoagulated patients or healthy volunteers, may also be appropriate [101-103]. See Figure 8 for an overview of the currently available data on this matter. What became clear from this limited number of studies is the variability in assay readout between different NOACs and reversal agents. With PCC, e.g., partial correction of TGA-peak was observed for rivaroxaban and apixaban, while complete parameter correction was observed with dabigatran [32,68]. Similarly, complete correction of the PT by PCC was observed for apixaban while PT correction was only partial for rivaroxaban and dabigatran [32,68]. The APTT was insensitive to NOAC reversal by PCC, while partly corrected by rFVIIa and activated PCC [102]. The Figure 8 summary also suggests general applicability of the TGA-AUC in monitoring NOAC reversal by PCC as well as by activated PCC (FVIIa containing PCC). TGA-AUC was not affected by rFVIIa. For this reversal agent, TGA-lag time seems to be the best option.

In summary, the PT/INR remains the assay of choice to monitor reversal of VKA anticoagulation. TGA and TEG/TEM may be useful in this respect, but this needs further exploration. TGA-AUC may be general applicable in monitoring NOAC reversal by PCC and activated PCC, but not by rFVIIa. TGA-lag time seems the most appropriate assay readout for the assessment of NOAC anticoagulation by rFVIIa, but again, this needs further validation.

#### (Pre)analytical conditions that affect the assessment of NOAC reversal

Monitoring in vivo NOAC reversal by non-specific prohemostatic agents (PCC, activated PCC, rFVII) remain a controversial issue, this despite growing evidence that these non-specific reversal agents are able to correct, at least in part, NOAC-induced hemorrhage (reviewed in: [28,104,105]). In rivaroxaban-anticoagulated human volunteers, e.g., the PT normalized completely upon treatment with PCC [106]. In contrast, PT correction was only partial in rivaroxaban-anticoagulated animals receiving PCC [107,108]. In PT, extent of reversal is dependent on NOAC concentration, NOAC type, PCC dose and used thromboplastin reagent [32,68]. These variabilities make it extremely difficult to compare PT outcome from different in vivo reversal studies.

When applying TGA-AUC as readout parameter for NOAC reversal by PCC, several analytical considerations must be taken into account. E,g., the correlation between TGA-AUC and PCC dose is non-linear and for rivaroxaban the curves are less steeper and show faster decay than for VKA (Figure 7). This decaying relationship was also observed for apixaban and dabigatran and confirm results from an earlier study on rivaroxaban [32,68]. At very high NOAC concentration (e.g. at 800 μg/l rivaroxaban in Figure 7), this non-linear relationship may result in an AUC never reaching 100%. Pertinent to this view is the observation in dabigatran-anticoagulated rats, showing normalization of TGA-AUC at low (200 μg/l) but not at high (1000 μg/l) dabigatran levels [73]. 

Another complicating factor in monitoring reversal of NOAC anticoagulation by TGA is that the amount of PCC required for AUC normalization depends on the in the assay used TF concentration. For rivaroxaban e.g., at 1 pM TF, TGA-AUC was reduced to 11% of normal by 200 μg/l rivaroxaban and PCC up to 4 IU/ml was unable to completely normalize the AUC. At 5 pM TF and the same rivaroxaban concentration, an AUC of 60% could be normalized with 1.2 IU/ml PCC, while at 20 pM TF a slight reduced AUC (84% of normal) required only 0.2 IU/ml PCC [68]. A similar observation was made for apixaban [32]. In contrast, in vitro reversal of dabigatran anticoagulation by PCC appeared TF concentration independent [32]. For the potential applicable reversal agents rFVIIa and activated PCC, any TF dependency remains to be established. The TF concentration dependency in monitoring reversal of rivaroxaban and apixaban induced anticoagulation by PCC, however, highlights the need for assay standardization.

There are several other (pre)analytical conditions to consider. Of potential importance are compositional differences between clotting factor concentrates, including the presence of heparin, that may translate into poor laboratory outcome while hemostatically effective [73]. Also the influence of blood cells and platelets on the PCC dose required for TGA normalization is an issue that needs further investigation [68].

#### Specialty tests, a pitfall in the assessment of NOAC reversal

Lack of awareness of the applicability of a certain laboratory test in monitoring OAC reversal has led to confusing recommendations. E.g., based on the outcome of the thrombin time (TT) and ecarin clotting time (ECT) in the reversal of NOAC anticoagulation by PCC in healthy volunteers, PCC was discarded as reversal agent for dabigatran while effective as a hemostatic drug in dabigatran-anticoagulated animals [73,106,109]. Indeed, TT and ECT are extremely sensitive to dabigatran anticoagulation [86]. However, these tests are insensitive to anticoagulation reversal by PCC. In the TT test, excess thrombin is added to a plasma sample, as such overruling the complete coagulation cascade (see Figure 2). As a consequence, dabigatran in the plasma sample will inhibit the added thrombin without being affected by increased clotting factor levels due to PCC administration. In the ECT test, all prothrombin in the plasma sample is converted to thrombin by the addition of the viper venom Ecarin. The ECT is only sensitive to prothrombin levels below 60% [110,111], a concentration not to be expected in NOAC treated individuals. Clotting time in the ECT test, like in the TT test, is prolonged by dabigatran present in the plasma sample, while an increase in vitamin K-dependent clotting factors upon PCC administration will be unnoticed. Also the diluted thrombin time, a test particularly suitable for dabigatran measurements, is not able to reveal reversal of dabigatran anticoagulation [73,112]. Similarly, chromogenic anti-Xa assays suited for rivaroxaban determinations, do not reveal reversal of anticoagulation by clotting factor concentrates. Global coagulation tests, measuring the complete hemostatic potential of a whole blood or plasma sample, are the only applicable tests for the determination of anticoagulation reversal by non-specific prohemostatic agents.

## Conclusion

While the global coagulation tests PT and APTT have been extensively studied for their applicability in measuring VKA as well as NOAC anticoagulation, comprehensive validation studies for TGA and above all for TEG/TEM are scare. Applicability of a particular test in monitoring NOAC or VKA anticoagulation is not translated directly to applicability in reversal assessment (Table 3). This review clearly shows that mPT (modified PT), TGA-lag time and TEG/TEM-R/CT are the most sensitive global assay parameters to assess NOAC anticoagulation. Although these time-based parameters do reveal VKA-reversal by PCC, they seem to be not suitable for assessing NOAC reversal by PCC and activated PCC. TGA-lag time, on the other hand, may be used to assess NOAC reversal by rFVIIa. This review also shows the potential usefulness of the TGA-AUC in monitoring reversal of VKA as well as NOAC anticoagulation by PCC. Analytical considerations, among others, are the influence of TF concentration and the presence of blood cells and platelets on the assay outcome. The poor sensitivity of current available global coagulation assays towards NOACs, together with an assay reagent- and NOAC concentration dependent anticoagulation reversal by PCC, explains the reported controversial data on the clinical usefulness of PCC as reversal agent for NOAC anticoagulation. As a final remark: global assays may only be used in animal studies and in clinical practice after extensive in vitro validation, an approach often neglected.

**Table 3:** **Applicability of laboratory assays in the management of oral anticoagulation**

	**Monitoring VKA treatment**		**Monitoring NOAC treatment**	
	**Anti-coagulation**	**Reversal**	**Anti-coagulation**	**Reversal**
Global assays				
APTT	P^1^	P^1^	P^1^	N^2^
PT/INR	A	A	P^1^	P^3,4^
TEG/ROTEM	Q^3^	Q^3^	P^1^	P^3^
TGA	A^3^	A^3^	P^1^	P^3,4^
Specialty assays				
ECT/TT	N^2^	N^2^	A	N^2^
Xa-i/DTI	N^2^	N^2^	A	N^2^

A, applicable.

P, partly applicable; use with caution.

Q, questionable.

N, not applicable.

^1^moderate to low sensitivity.

^2^no sensitivity.

^3^requires further validation and standardization.

^4^assay normalization depends on NOAC type, NOAC concentration, TF concentration and used thromboplastin reagent.

This table summary clearly shows that the applicability of a particular test in monitoring NOAC or VKA anticoagulation does not translate directly to applicability in reversal assessment. The PT remains the appropriate test when managing VKA anticoagulation. TGA may also be suitable, but this requires further validation. Specialty assays suited for NOAC monitoring (ecarin clotting time, ECT; thrombin time, TT; direct thrombin inhibitor assay, DTI; chromogenic factor Xa inhibitor assay, Xa-i) do not apply to reversal assessment. Monitoring NOAC reversal is feasible with PT, TEG/ROTEM and TGA. However, most readout parameters only show partial NOAC reversal. Global assays, in general, show low sensitivity to NOACs (see Table 2).
